# Betulinic acid is a PPARγ antagonist that improves glucose uptake, promotes osteogenesis and inhibits adipogenesis

**DOI:** 10.1038/s41598-017-05666-6

**Published:** 2017-07-18

**Authors:** Gloria Brusotti, Roberta Montanari, Davide Capelli, Giulia Cattaneo, Antonio Laghezza, Paolo Tortorella, Fulvio Loiodice, Franck Peiretti, Bernadette Bonardo, Alessandro Paiardini, Enrica Calleri, Giorgio Pochetti

**Affiliations:** 1 Dipartimento di Scienze del Farmaco, Università degli Studi di Pavia, Via Taramelli 12, 27100 Pavia, Italy; 2 Istituto di Cristallografia, Consiglio Nazionale delle Ricerche, Via Salaria Km. 29, 300, 00015 Monterotondo Stazione, Roma Italy; 3 Dipartimento di Farmacia-Scienze del Farmaco, Università degli Studi di Bari “Aldo Moro”, Via E.Orabona 4, 70126 Bari, Italy; 4 Inserm UMR 1062, Faculté de Médecine Timone, Aix-Marseille University, 27 bd Jean Moulin, 13385 Marseille, France; 5 Department of Biology and Biotechnology, Università “La Sapienza” di Roma, via dei Sardi 70, 00185 Roma, Italy

## Abstract

PPAR antagonists are ligands that bind their receptor with high affinity without transactivation activity. Recently, they have been demonstrated to maintain insulin-sensitizing and antidiabetic properties, and they serve as an alternative treatment for metabolic diseases. In this work, an affinity-based bioassay was found to be effective for selecting PPAR ligands from the dried extract of an African plant (*Diospyros bipindensis*). Among the ligands, we identified betulinic acid (BA), a compound already known for its anti-inflammatory, anti-tumour and antidiabetic properties, as a PPARγ and PPARα antagonist. Cell differentiation assays showed that BA inhibits adipogenesis and promotes osteogenesis; either down-regulates or does not affect the expression of a series of adipogenic markers; and up-regulates the expression of osteogenic markers. Moreover, BA increases basal glucose uptake in 3T3-L1 adipocytes. The crystal structure of the complex of BA with PPARγ sheds light, at the molecular level, on the mechanism by which BA antagonizes PPARγ, and indicates a unique binding mode of this antagonist type. The results of this study show that the natural compound BA could be an interesting and safe candidate for the treatment of type 2 diabetes and bone diseases.

## Introduction

Peroxisome proliferator-activated receptors (PPARs) are a group of transcription factors belonging to the nuclear receptor superfamily. This group has emerged as a key player in the regulation of important metabolic pathways and cellular functions in the pathophysiology of diabetes, obesity, and in the related cardiovascular and cerebrovascular complications^1, 2^. As such, the three PPAR subtypes, designated α, γ, and δ, bind to fatty acids and their metabolites, and consequently regulate the expression of genes involved in the transport, metabolism and buffering of these ligands within cells^1, 3–6^. The thiazolidinedione (TZD) anti-diabetic agents (e.g., rosiglitazone and pioglitazone) are PPARγ agonists whose insulin-sensitizing actions are largely mediated by pleiotropic effects in adipose tissue^5–8^, whereas the fibrate anti-atherosclerotic, hypolipidaemic agents (e.g., fenofibrate and gemfibrozil), are PPARα agonists^5, 6, 9, 10^. Despite their widespread prescription, PPAR-activating drugs have unwanted effects that cannot be underestimated^11, 12^. To overcome these side effects, novel synthetic PPAR ligands have been identified. These include PPARα/γ dual agonists or PPARα/γ/δ pan-agonists, which beneficially alter carbohydrate and lipid metabolism in a coordinated manner, and selective PPARγ modulators (SPPARγMs) with robust anti-diabetic efficacy and fewer adverse effects than currently available agonists. Nonetheless, the final candidate in late-stage trials, the dual PPARα/γ agonist aleglitazar, has been withdrawn, owing to its toxicity and lack of efficacy, and only a small number of remaining candidates are in Phase II trials. For this reason, there is a need to discover new classes of compounds that better satisfy the criteria of therapeutic efficacy with decreased side effects. In this regard, a new class of potent PPARγ antagonists has recently been developed^13–16^ (bexarotene, 2-phenylamino pyrimidine and N-biphenylmethylindole derivatives). These antagonists maintain a robust antidiabetic activity in rodent models of diabetes and may provide a safe alternative to targeting PPARγ for the therapeutic intervention in insulin resistance and type-2 diabetes.

Given the structural diversity of PPAR ligands, research efforts to identify unknown PPAR modulators as novel drug candidates may be extended through screening of plant extracts^17, 18^.

Among the research strategies for the discovery of new pharmacologically active plant compounds, the ethnopharmacological approach, that is, the therapeutic use of popular tradition plants, serves as a basis for the selection of new material subjected to pharmacological assays.

Peculiarly, African medicinal plants has not been studied as fully as Indian and Chinese treatments. In this context, an ethnobotanical survey was conducted by our research group among the pygmies Baka, a community living in the south of Cameroon and possessing a consolidated vernacular knowledge in the field of traditional medicine through the use of medicinal plants. Some of these medicinal plants were selected as candidates to validate their traditional use^19–23^. Among them, *Diospyros bipindensis* was identified as the most interesting plant for possible activity on PPARs receptors. In fact, some plants of the same family and genus are known to have anti-inflammatory, anti-diabetic and hypoglycaemic properties^24–30^. *Diospyros bipindensis* has been studied, and some secondary metabolites have been purified and identified including plumbagin, betulinic acid, caniculatin, 4-hydroxy-5-methyl-coumarin and ismailin^22^ (Figure S1 of Supplementary Information). Interestingly, it has recently been reported that plumbagin, isolated from Plumbago zeylanica root, has antidiabetic activity in streptozotocin-induced diabetic rats by increasing insulin secretion^31^. Moreover, betulinic acid (BA), a naturally occurring plant-derived triterpenoid present in many fruits and vegetables, has been shown to have a wide variety of pharmacological and biochemical effects, including anti-inflammatory and anticancer activities and inhibition of adipogenesis in mice fed a high-fat diet^32–39^. In this study, an assay based on PPARγ affinity of potential ligands from a dried plant extract of *Diospyros bipindensis* was developed to perform preliminary screening and ranking of binding partners. The histidine-tagged protein was immobilized on HIS-Select^®^ Spin Columns solid-phase extraction cartridges^40–42^. Then, the affinity-cartridges were tested for their ability to fractionate the components of a dichloromethane extract (DME) of *Diospyros bipindensis* on the basis of the biological interactions with the immobilized target, thus providing an affinity ranking. All the compounds were further analysed for their *in vitro* activity (agonist and/or antagonist assay) towards PPARα and PPARγ receptors by using GAL4-PPAR transactivation assay. Plumbagin was the only ligand showing PPARγ partial agonism, whereas PPARα transactivation activity was not observed in response to any ligand. Displacement experiments showed that BA competes with rosiglitazone in PPARγ, and Wy-14643 in PPARα, with micromolar IC_50_ values. Interestingly, BA was also the ligand with the highest affinity. Subsequently, X-ray studies were performed on the complex between PPARγ and BA and a possible structural mechanism by which BA antagonizes PPARγ is proposed. Finally, we found that BA increases basal glucose uptake in 3T3-L1 adipocytes, inhibits adipogenesis, decreases the expression of adipogenic genes, and promotes osteogenesis, thus confirming the already known properties of this natural compound as a potential candidate for the treatment of bone diseases and type 2 diabetes, with potentially decreased side effects compared with those of TZDs.

## Results and Discussion

### PPARγ Immobilization

The procedure described in *Methods* allowed a high protein loading, thus resulting in the immobilization of approximately 250 μg of PPARγ nuclear receptor (immobilization yield ≈ 38%), as estimated by Bradford assays on the receptor solution, before and after immobilization, and on the washing fraction. Non-specific interaction between ligands and the silica support was measured through a control support which was prepared through the same method, except that no receptor was added during the immobilization step. This control material was washed and stored in the same manner as the immobilized PPAR support.

### Bioaffinity experiments with known ligands

To demonstrate that immobilized PPARγ recognizes ligands according to their affinity, three known ligands endowed with different potency (compounds A, B and C) were selected^43–45^. The structure of these compounds, together with their EC_50_ and E_max_, are shown in Figure S2.

The samples were loaded alone and in mixture on the control and receptor cartridges (CC and RC, respectively) and the data were processed according to the protocol described in *Methods*. In these experiments, the assumption was that the non-specific interactions between the support and the compound under study were the same for the control and receptor columns.

If a delay in the elution time on the experimental column was observed, the difference provided a relative measurement of the extent of specific interactions between the injected compound and the immobilized receptor. In this way, ranking a set of compounds according to affinity was achieved.

The experimental breakthrough elution profiles for the three analytes on RC and CC, which eluted independently, are shown in Figure S3. The same experiment was run with the three analytes in mixtures and in equimolar concentration.

For each analyte, a Δv shift value was derived. The Δv shift was the elution volume difference measured at 50% of elution on the sigmoidal fronts obtained on RC and CC columns. Δv shift values are reported in Figure S3. From these data, it was clear that a specific interaction with the receptor occurred. The rank order followed the general trend of EC_50_. The ligand affinity was classified according to the Δv shift value, for which higher values correspond to the analytes with higher affinity (compound C).

Notably, the same rank order was obtained with the three analytes in mixture, but with lower Δv shift values. This result was not surprising, because competitive displacement mechanisms during the affinity process cannot be excluded. Together, these results demonstrated that the receptor maintains its binding properties and that the cartridges can be used as a qualitative method to discriminate among low, medium and high-affinity ligands.

### Bioaffinity experiments on DME and single analytes

This result prompted us to investigate the ability of the affinity-based assay to rank compounds in the dried plant extract.

**Figure 1:**
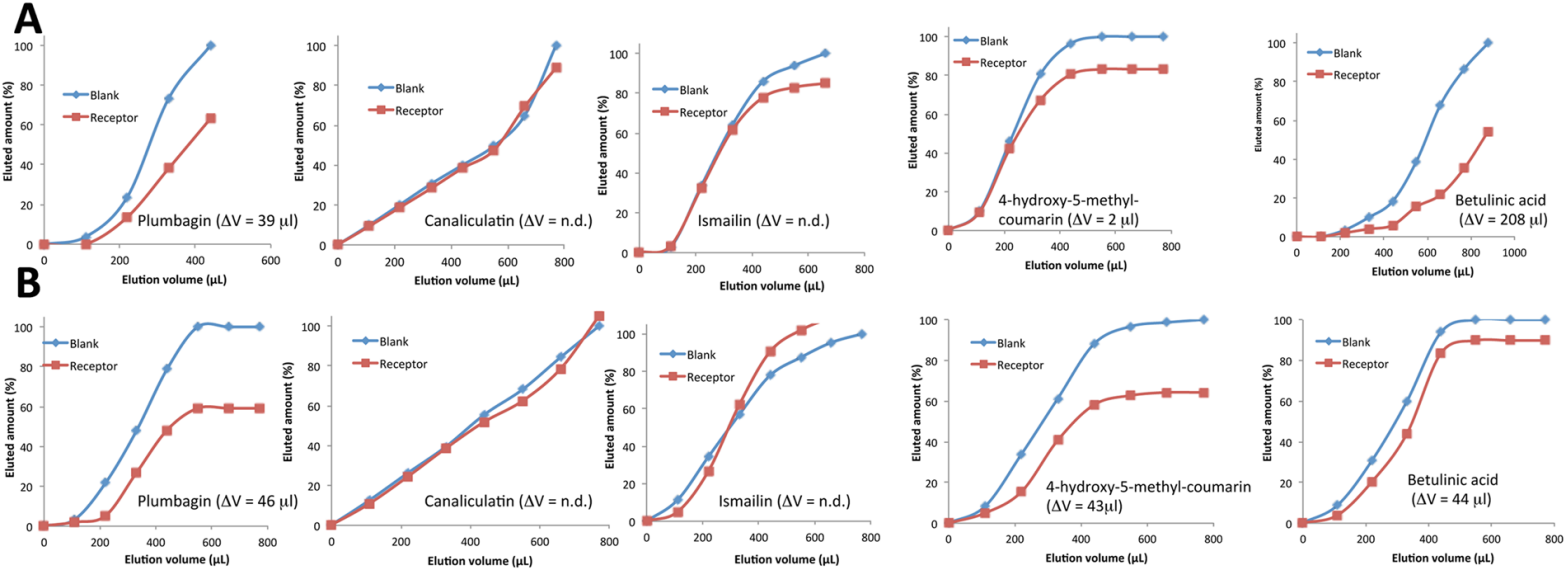
Breakthrough profiles of the five analytes. Elution profiles (percentage of total eluted analyte) of the five secondary metabolites of *Diospyros bipindensis* on receptor and blank cartridges, together with the calculated Δv shift: Elution profile of (**A**) the single analytes, and (**B**) the analytes eluted in mixture.

A comparison of breakthrough profiles for the main constituents of *Diospyros bipindensis* as a mixture and alone on RC and CC is shown in Fig. 1.

The analysis of the ligands in the DME indicates that only three ligands (i.e., plumbagin, BA and coumarin) interact with the receptor (Fig. 1B). In contrast, the same elution profile was obtained on RC and CC for ismailin and caniculatin, whose two breakthrough profiles overlapped.

The same experiments were carried out with the isolated ligands (Fig. 1A) and the results were confirmed for all of them, except for coumarin, which did not exhibit any detectable interaction. The high Δv shift observed for betulinic acid was ascribed to the low concentration used in this experiment (1 μg/ml). The Δv shifts of the five analytes are reported in Fig. 1.

### BA is an antagonist of rosiglitazone

The five compounds were evaluated with luciferase-based transactivation assays to determine their agonist activity towards the human PPARγ and PPARα subtypes. HepG2 cells were transiently transfected with either the GAL4-PPARα or the GAL4-PPARγ chimeric construct, together with the luciferase reporter vector containing the pGal5TkpGL3 constructs, and then treated with each ligand at two concentrations (5 μM and 25 μM). Rosiglitazone (2 μM) and Wy-14643 (10 μM) were used as reference compounds for PPARγ and PPARα transactivation potentials, respectively. The maximum fold induction obtained with the reference agonist was set at 100%.

**Figure 2:**
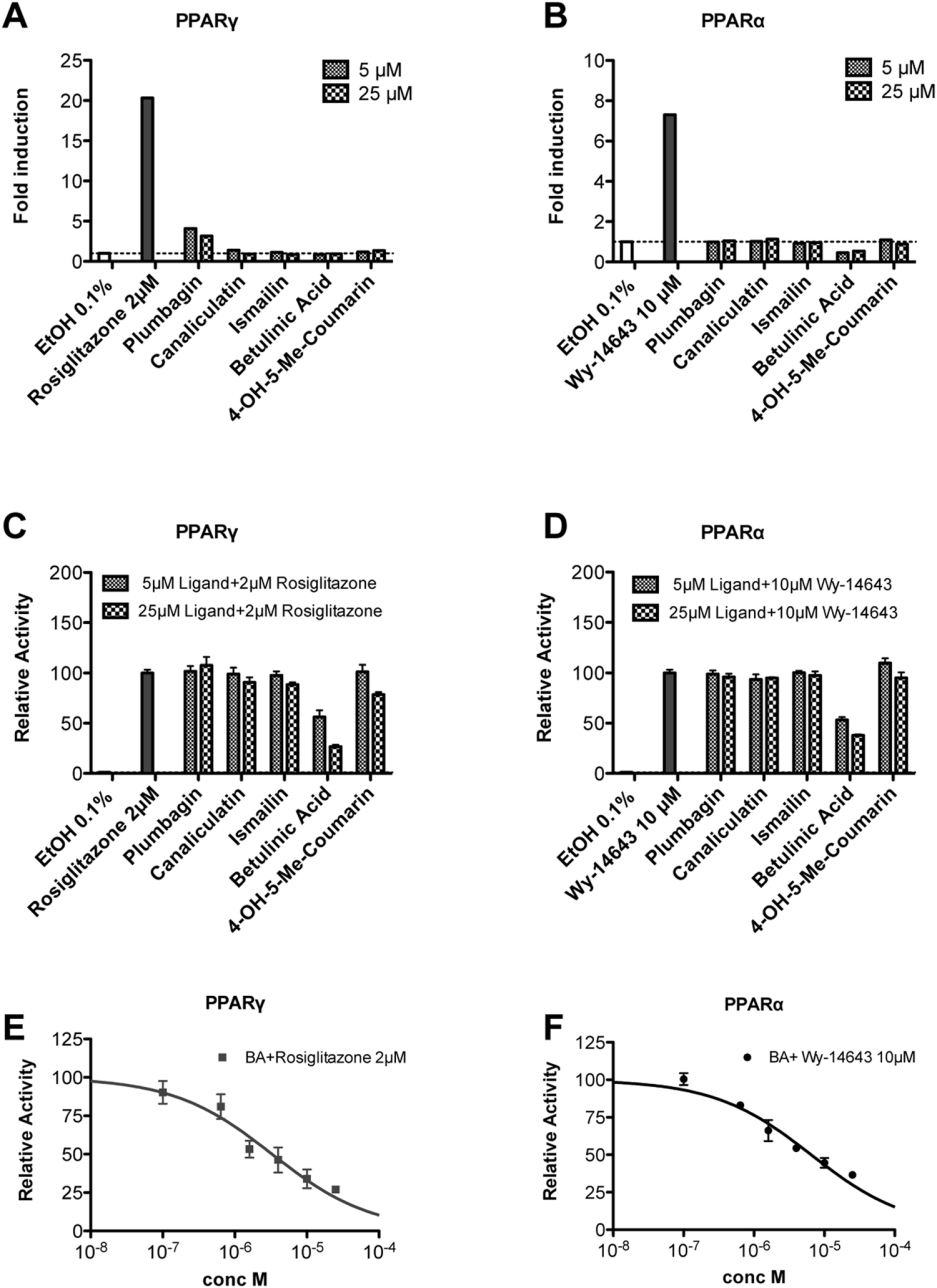
Transactivation assay and antagonist effect of the five ligands towards PPARγ and PPARα in HepG2 cells. Fold induction at two concentrations (5 and 25 μM) of the five compounds over vehicle (EtOH, 0.1%) on (**A**) PPARγ and (**B**) PPARα as determined by luciferase-based transactivation assays. Reference compounds: rosiglitazone (2 µM) and Wy-14643 (10 µM). Plumbagin was the only PPARγ partial agonist in the series (Emax = 19 ± 2% compared with rosiglitazone), and the others were inactive. For plumbagin, it was not possible to calculate EC_50_, because of its cytotoxicity starting from 25 µM. No PPARα transactivation was observed in response to any ligands. The antagonist effect of the five ligands on (**C**) PPARγ and (**D**) PPARα was measured at two concentrations (5 and 25 μM) on Gal4–hPPARγ LBD or Gal4–hPPARα LBD transfected cells treated with rosiglitazone at 2 µM or Wy-14643 at 10 µM. Data from luciferase reporter assays indicated that only BA was able, at increasing concentrations, to displace (**E**) rosiglitazone from PPARγ and (**F**) Wy-14643 from PPARα with IC_50_ values of 3.1 ± 1.2 μM and 7.4 ± 1.8 μM, respectively. All data are normalized with respect to control, and values are presented as the means ± SEM of n = 2 experiments performed in triplicate.

Plumbagin was the only PPARγ partial agonist of the series (Emax = 19 ± 2% compared with rosiglitazone), and all the other compounds were inactive (Fig. 2A). It was not possible to calculate the EC_50_ for plumbagin, because of its cytotoxicity starting from 25 μM. No PPARα transactivation was observed in response to any ligand (Fig. 2B).

The demonstration that the compounds other than plumbagin have affinity towards PPARγ (results from bioaffinity experiments) but do not activate it (transactivation assay), led us to investigate whether these compounds may exhibit PPAR antagonist activity. To this aim, Gal4–hPPARγ LBD or Gal4–hPPARα LBD transiently transfected HepG2 cells were co-incubated with or without ligand in the presence of either rosiglitazone or Wy-14643 for 20 h. Data from luciferase reporter assay indicated that only BA was able to displace rosiglitazone from PPARγ, and Wy-14643 from PPARα, with IC_50_ values of 3.1 ± 1.2 µM and 7.4 ± 1.8 µM, respectively (Fig. 2C–F).

### Isothermal titration calorimetry

ITC reverse titration experiments were performed to further confirm the interaction of BA with PPARγ. Reverse titration ensured a 10-fold molar excess in ligand to protein at the beginning of the experiment. In this way, it was possible to determine a binding stoichiometry also different from 1:1. The results (Figure S4) indicated that only one molecule of BA binds to the PPARγ ligand binding domain (LBD) with a K_d_ of 4 μM.

### PPARγ/BA crystal structure

X-ray diffraction data were collected for the complex PPARγ/BA to provide an explanation at the molecular level for the antagonist character of BA towards PPARγ.

The statistics of crystallographic data and refinement is summarized in Table 1.

**Table 1:** Statistics of Crystallographic Data and Refinement.

	PPARγ/BA
Space group	*C2*
Wavelenght (Å)	0.9537
Temperature (K)	100
Cell axes (Å)	92.86; 60.85;118.03
Beta angle (°)	102.482
Resolution range (Å)	50.00–2.00 (2.20–2.00)
R_merge_ (%)	2.7 (41.3)
Unique reflections	43046
I/σ(I)	20.33 (2.63)
CC(1/2)	100.0 (84.1)
Completeness (%)	98.6 (97.6)
R_factor_ (%)	22.7
R_free_ (%)	26.4

*The values in parentheses refer to the outer shell.

**Figure 3:**
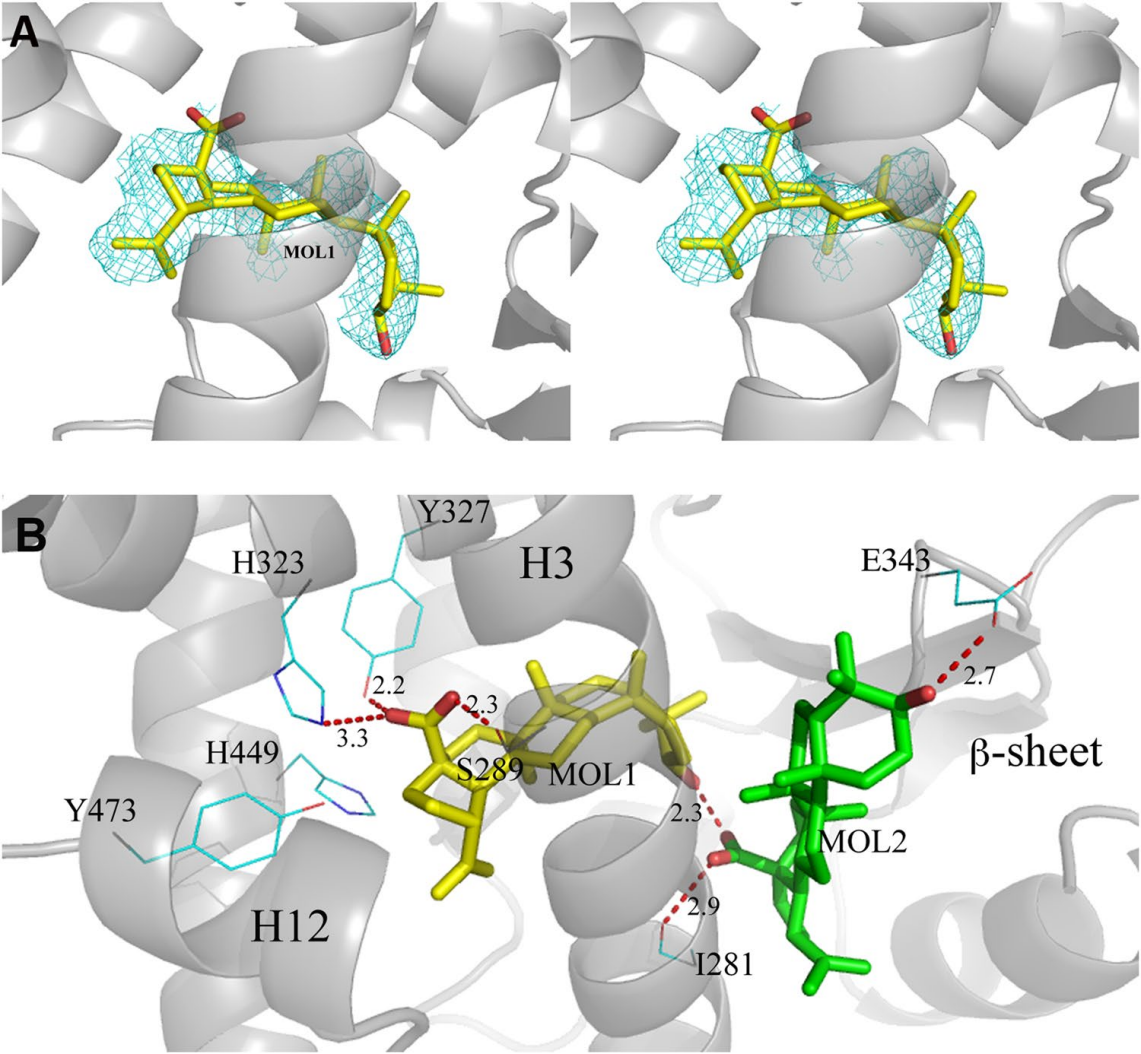
BA interactions with the LBD of PPARγ. (**A**) 2Fo-Fc omit map around molecule 1 of BA (yellow) is shown in mesh and contoured at 0.7 σ; (**B**) H-bond interactions (red dashed lines) of molecule 1 (yellow) and the docked molecule 2 (green) of BA with residues of the LBD of PPARγ.

There are two independent protein molecules in the asymmetric unit (chain A and B). In chain A, there is clear density in the region usually occupied by PPARγ full agonists where it was possible to fit a single molecule of BA (Fig. 3A).

Additionally, less evident electron density was also observed in the region usually occupied by partial agonists, between the helix 3 (H3) and the β-sheet, but any attempt to fit a second BA molecule into the electron density did not succeed. For this reason, we decided to perform docking simulations with the software Molegro to confirm the position of the first molecule in the primary site and to investigate the possibility that a second BA molecule might occupy an alternate secondary site. The simulations confirmed the occupation of the primary site (first pose in the ranking) with the docked ligand in almost the same position, as refined in Phenix (Figure S5). Once fixed the position of the first molecule in the primary site, a set of energetically favourable poses of a second molecule of BA in the secondary site was obtained (Figure S6). The first BA molecule has all its methyl groups engaged in vdW contacts with the protein environment (L453, F282, M364, C285, L330, V339). The carboxylate group makes H-bonds bridging the hydroxyl moieties of Y327 and S289. One of the two oxygen atoms is also engaged in a H-bond with the side-chain of H323. The 3-OH group of BA is involved in a H-bond with the carboxylate group of the second docked BA molecule. In Fig. 3B the interactions (H-bonds) of both molecules of the ligand (MOL1 and the docked MOL2) in the LBD of PPARγ are shown. Other structures of PPARγ complexes with two simultaneously bound molecules are present in the pdb (pdb codes 2vsr, 3adw, 3k8s and 4e4k)^46–49^, and we compared their structure with that of PPARγ/BA (Figure S7). As shown in the Figure, whereas the ligands similarly occupy the primary canonical site, different binding modes are observed for the second molecule bound to the alternate site, in a wide region extending from the ω-loop, at the entrance of the pocket, until the arm II.

### H-bond network involving the triad Y474, H323 and H449

**Figure 4:**
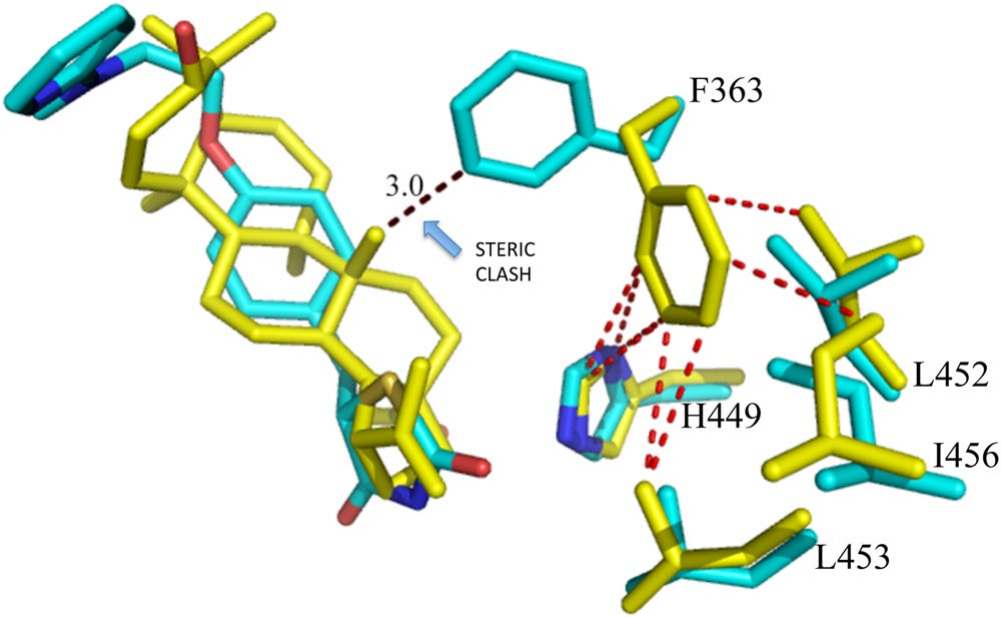
Superposition of PPARγ/BA and PPARγ/rosiglitazone complexes. Superposition of the crystal structures of the PPARγ/BA (yellow) and PPARγ/rosiglitazone (pdb code 2PRG) (cyan) complexes. The F363 side-chain in gauche conformation (cyan) would sterically clash (black dashed lines) with a methyl group of BA. The gauche* conformation of F363 allows vdW interactions (ranging from 3.3 to 3.7 Å) with hydrophobic residues on H11 (red dashed lines).

Unlike the strong agonist rosiglitazone (PDB code 2PRG), BA does not interact directly with Y473 by H-bonds through its carboxylate group, which instead is engaged in H-bonds with Y327, H323 and the side-chain of S289 (Fig. 3B). In contrast, BA contacts the Y473 OH by vdW interactions through a carbon atom of its 5-membered ring (shortest C-O distance: 3.1 Å). This contact makes the active conformation of the helix 12 less stable, as denoted by a comparison of the H12 B factors with those of the structure with the full agonist rosiglitazone (pdb code 2PRG) (Figure S8). This Figure shows a dramatic decrease of the average B factors in proximity of Y473, owing to the H-bond with rosiglitazone, which it is not present in the PPARγ/BA structure. The H-bond network involving the triad Y473, H323 and H449 is less stable than that realized by full agonists such as rosiglitazone, as denoted by the slightly shifted position of the activation function-2 helix (AF2 or H12) from its active conformation, with the consequent loss of the H-bond between the H449 ring and the Y473 OH (distance of 3.6 Å). From the crystal structure, a steric clash is seen between carbon atoms of the ligand and the ring of H449 (shortest distance 2.9 Å). Moreover, the environment around H449 is different than that of the complex with rosiglitazone, because the F363 side-chain changes its conformation owing to a steric clash with one methyl of BA (3.0 Å), and approaches H449 (3.3 Å), L453 (3.6 Å), L452 (3.7 Å), and the methyl group of I456 (3.5 Å) which is slightly displaced from its original position (Fig. 4).

Therefore, the dynamics of the helix 11 (H11) around H449 is changed and may affect the stability of H12. In the structure of PPARγ with the previously reported antagonist SR1664, a loss of stabilization of the region of H11 near H449 has also been observed in HDX experiments^50^. We suggest that the antagonist character of BA is due to the loss of direct H-bonds with Y473 and the consequent destabilization, or sub-optimal stabilization, of H12 and H11.

The micromolar affinity (K_d_ = 4 μM) of BA for the LBD of PPARγ, as determined by ITC (Figure S4), was sufficient to displace rosiglitazone, as observed in the competitive test (Fig. 2E).

### Comparison with the antagonist SR1664 and the agonist SR1663: the F282/F363 switch mechanism

**Figure 5:**
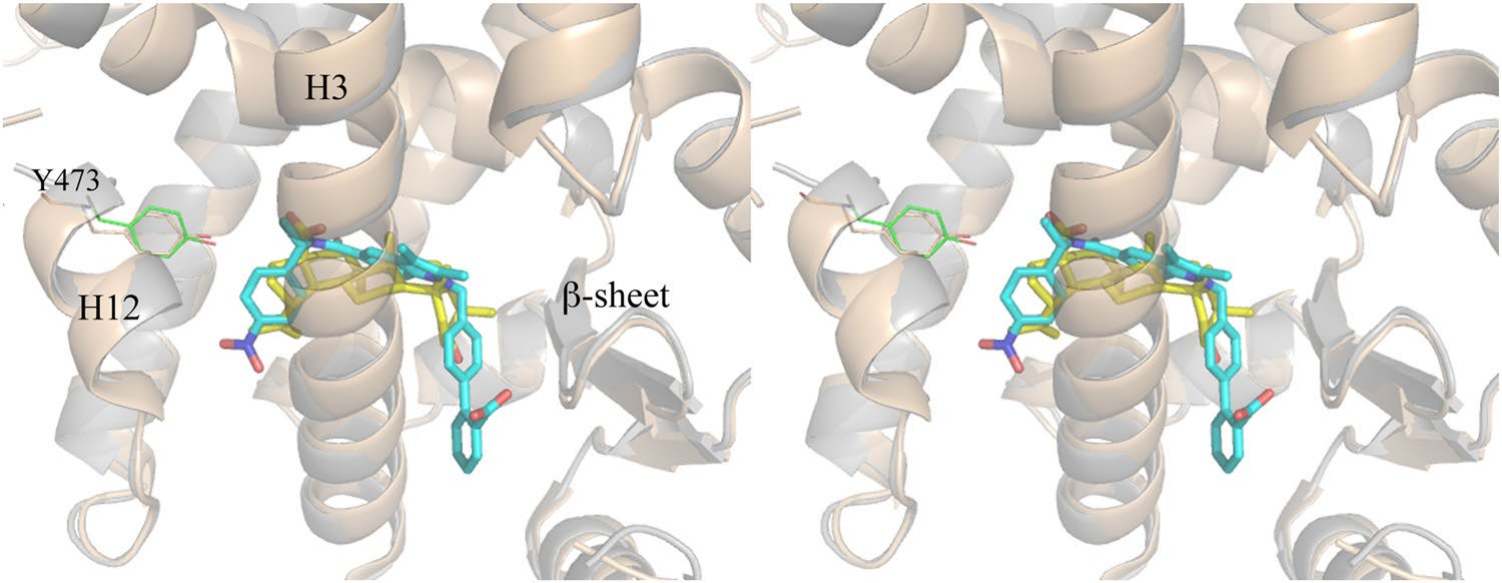
Superposition of PPARγ/BA and PPARγ/SR1664 crystal structures. Stereo view of the superposed crystal structures of PPARγ/BA (cartoon grey, BA yellow) and PPARγ/SR1664 complexes (cartoon wheat, SR1664 cyan).

It is interesting to compare the crystal structure of PPARγ/BA with that of the complex with another PPARγ antagonist. Figure 5 shows the superposition of the structures of BA and SR1664^13, 50^ (PDB code 4R2U) in complex with PPARγ.

**Figure 6:**
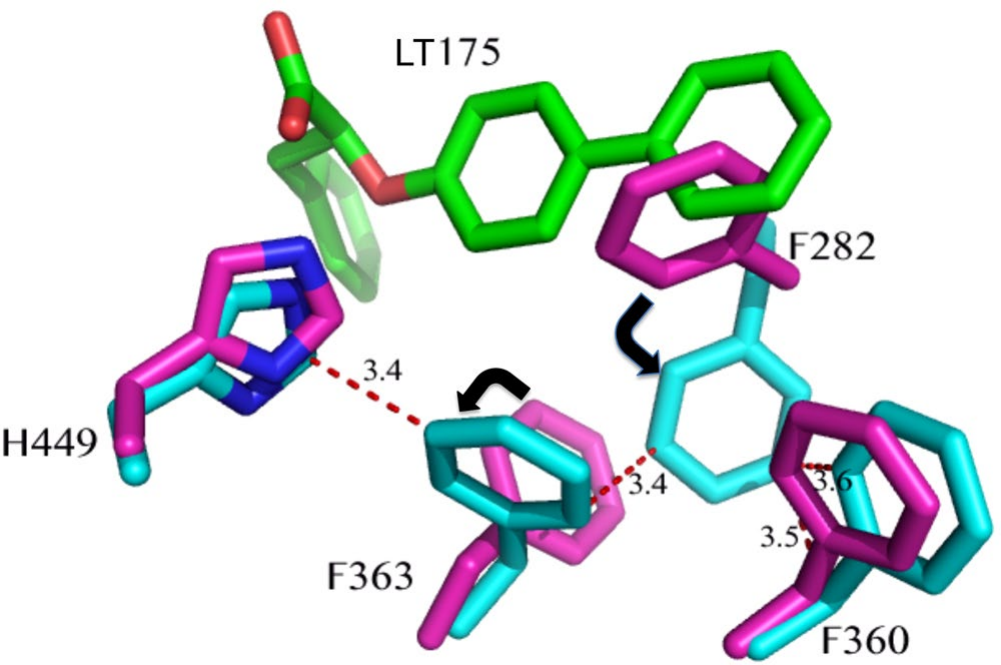
F282/F363 switch mechanism. In the PPARγ/LT175 X-ray structure (cyan) the ligand LT175 (green) forces the F282 side-chain to switch from the trans to the gauche* conformation, and in turn the F363 side-chain also changes its conformation by 180°, contacting H449. In the new conformation, the F282 aromatic ring makes favourable pi-stacking interactions with the rings of F360 and F363. Apo-PPARγ residues are in purple.

The two compounds occupy a similar position in the LBD, both contacting Y473 only through vdW interactions and instead forming a H-bond with Y327. In the case of the bulkier SR1664, the authors^13^ have observed steric clashes, not present in the *R*-enantiomer SR1663, with the F282 aromatic ring and hydrophobic side chains of H11, such as that of Leu 453, as well as that of Leu 465 of the loop 11–12. Therefore, the authors have suggested that SR1664 actively antagonizes PPARγ through a stereo-specific AF2-mediated, F282-dependent clash. Moreover, they have confirmed the role of F282 in the antagonism of SR1664, demonstrating that this ligand becomes an agonist after F282A mutation. However, the shortest distance of SR1664 with F282 is 3.0 Å (NO_2_-C), a result consistent with a strong vdW interaction rather than a steric clash. As discussed below, the F282A mutation may result in a different position of the ligand into the pocket, thus explaining its agonism towards the mutant. The corresponding *R*-enantiomer SR1663 shows agonist properties (80% efficacy compared to rosiglitazone). Observing the interactions of the ligand with the protein molecule A of PPARγ in which H12 is in the active conformation (H12 of the molecule B of the dimer is in an inactive conformation and it should not be considered to discuss its interaction with an agonist), a greater protruding of the NO_2_ group towards F282 is seen, owing to the different stereochemistry, which would result in a strong clash with F282 if this residue were to assume its usual conformation. In fact, there is poor electron density in the position of F282, and the authors were able to assign only the position of the Cβ atom of the F282 side-chain. However, a more accurate observation of the electron density maps reveals a possible different conformation of the F282 side-chain, involving switching from trans to gauche* (Figure S9). A similar situation has already been observed in the structure with the ligand LT175^51^ (PDB code 3B3K), in which the bulky and rigid diphenyl group of the ligand displaces the side-chain of F282, shifting in turn those of F363 and I456 and opening a new hydrophobic pocket in the LBD between H11 and H3 (Fig. 6).

This was also confirmed in the PPARγ/SR1663 structure by the shifted conformation of F363 with respect to that observed in the apo-PPARγ, and the alternate conformation of C285, which in this new conformation can interact with the side-chain of F282. This role of F282 as gate-keeper has also recently been observed in the PPARα complex with the ligand AL29-26^52^ (in this case the PPARα corresponding residue is F273). The accommodation of the nitro-substituted ring of SR1663 in the new pocket, as well as the diphenyl moiety of LT175, can determine the agonist character of the ligand with an additional stabilization of H11 and H3. It is possible that SR1664 in the F282A mutant can assume a similar deeper position in the pocket, becoming an agonist, but only its crystal structure in the complex with the mutant would be able to unveil this result.

The structures of the two compounds BA and SR1664, both lacking the H-bond interaction with Y473 on H12, together suggest that this feature might be responsible for the antagonist character of these compounds. In any case, also SR1663 shows no direct interactions, other than vdW contacts, with Y473, thus suggesting that a direct hydrogen bond with Y473 is not necessarily required for some agonist activity, but instead the stabilization of H12 in its active conformation may be more important, as well as the proper conformation of the other helices forming the AF2 surface. In the case of the antagonist BA, the weakening of the interaction with H12, associated with a different stabilization of H449 on H11, may be responsible for different dynamics of the helices forming the co-activator binding site, thereby determining the antagonist character of the ligand.

In conclusion, the PPARγ LBD is formed by closely related domains whose dynamics, after ligand binding into different regions of the LBD (delimited by H3, H12, H11 and the loop 11–12, as in the case of full agonists, or by H3, the β-sheet and the ω loop, as in the case of partial agonists), can produce long range allosteric effects affecting the recruitment of co-activators or the phosphorylation of S245 (S273 in PPARγ2), which is currently considered as one of the most important mechanisms in PPARγ activity modulation^50^.

### BA inhibits adipocyte differentiation and stimulates osteogenesis

PPARγ is a critical factor in the reciprocal regulation of adipogenesis and osteogenesis. Indeed, it has been shown that its decrease (inhibition or deficiency) limits adipogenesis and increases osteogenesis^53, 54^. To investigate the effect of BA on adipogenesis and osteogenesis, we used adipocyte (3T3-L1 cells) and osteoblast (MC3T3-E1) murine cell models of differentiation.

**Figure 7:**
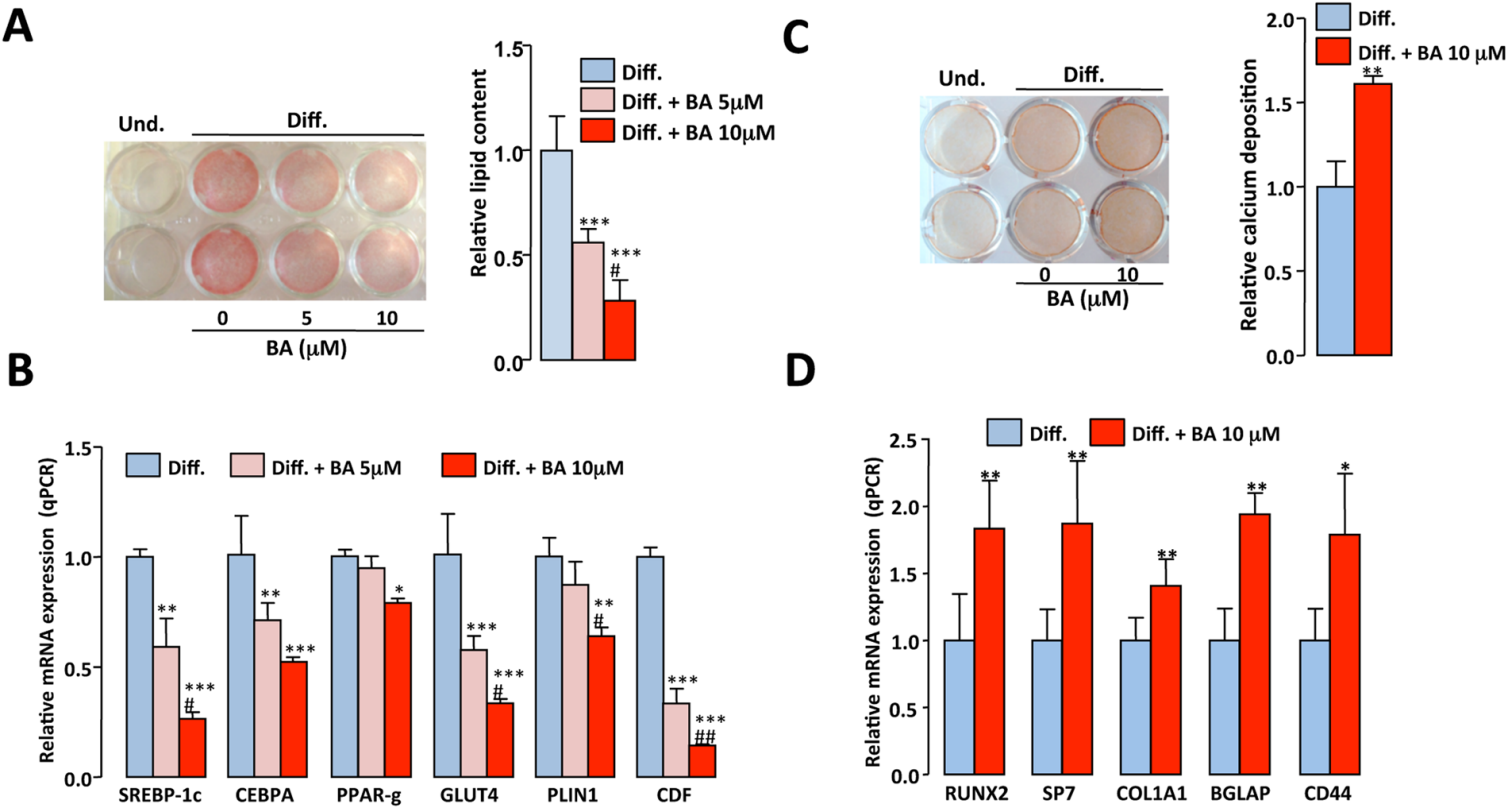
BA decreases differentiation of mouse preadipocytes and increases osteoblast differentiation. 3T3-L1 preadipocytes were induced to differentiate into adipocytes, then were treated with vehicle alone (DMSO) or betulinic acid (BA, 5 or 10 μM) for 4 days. (**A**) Lipids contained in undifferentiated (Und.) and differentiated (Diff.) cells were stained with Oil Red O (a representative image is shown) then bound dye was solubilized and quantified spectrophotometrically. Values are mean ± SD corrected for the mean value obtained with undifferentiated cells and expressed relative to the differentiated situation. (**B**) Expression levels of adipogenesis-related genes were measured by qPCR. Values are expressed as the means ± SD expressed as fold relative to differentiated (Diff.) situation. *P < 0.05, **P < 0.01, ***P < 0.001 vs. Diff.; ^#^P < 0.01, ^##^P < 0.01 vs. Diff. + BA 5 μM. (one-way ANOVA followed by Bonferroni post hoc test). MC3T3-E1 cells were cultured in osteogenic medium in the absence or presence of betulinic acid (BA, 10 μM) for 14 days. (**C**) Calcium deposition was identified by Alizarin Red S staining of undifferentiated (Und.) and differentiated (Diff.) cells (a representative image is shown), and then bound dye was extracted and quantified spectrophotometrically. Values are expressed as in Fig. 7A. (**D**) Expression levels of osteogenesis-related genes were measured by qPCR. Values are expressed as in Fig. 7B. *P < 0.05, **P < 0.01, vs. Diff.; (Unpaired t-test).

Differentiated 3T3-L1 cells accumulated intracellular lipids, as judged by their positive staining with Oil Red O (Fig. 7A left). The addition of BA after the induction phase of 3T3-L1 adipocyte differentiation dose-dependently decreased the Oil Red-O staining (Fig. 7A). BA also reduced the expression of key transcription factors involved in the early steps of adipogenesis (SREBP-1c, CEPBA and PPARγ) and that of differentiated adipocyte markers (GLUT4, PLIN, CDF) (Fig. 7B). These results indicate that BA treatment of preadipocytes inhibits adipogenesis.

Osteoblast differentiation of MC3T3-E1 cells was assessed by the staining of calcium deposition with Alizarin Red S (Fig. 7C left). The addition of BA to the osteogenic culture medium increased the calcification of bone cells (Fig. 7C) and the expression levels of early (RUNX2, SP7, COL1A1) and late (BGLAP, CD44) osteoblast markers (Fig. 7D), thus indicating that BA increases osteogenesis.

### BA improves the basal glucose uptake

**Figure 8:**
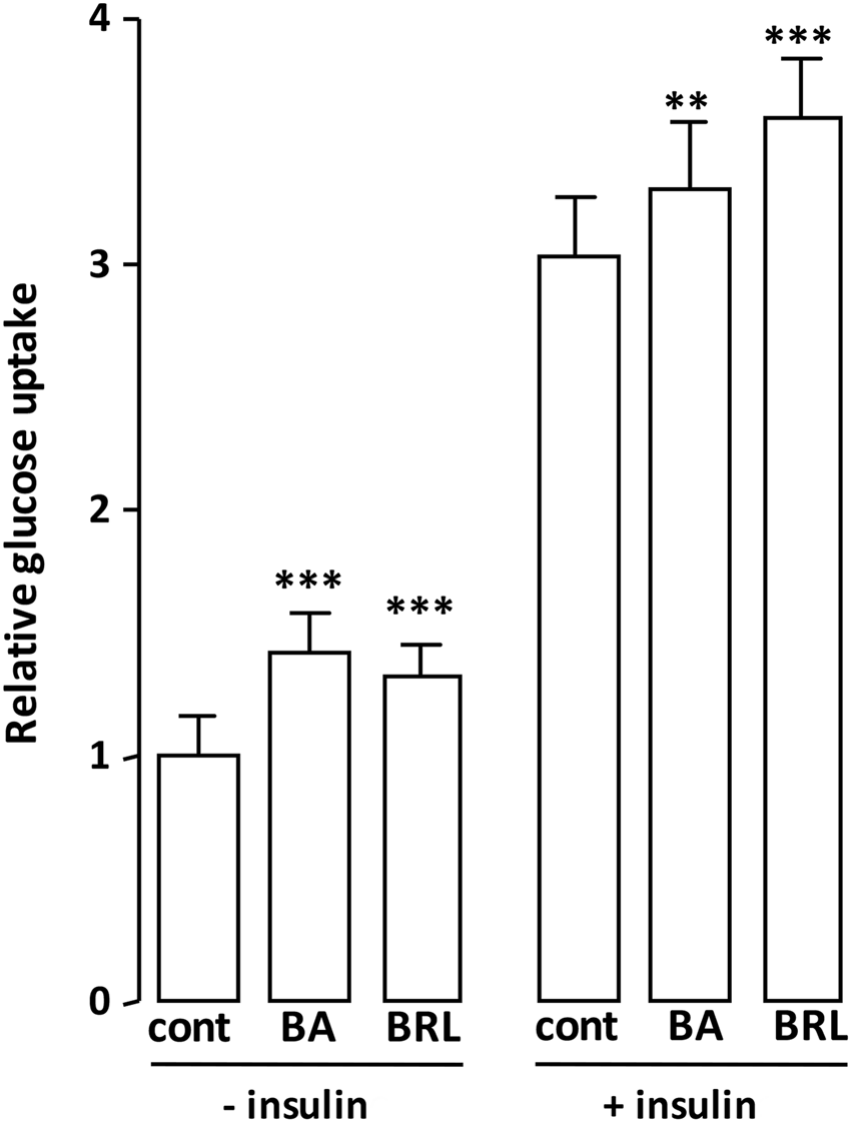
Cellular glucose uptake rate upon exposure to BA. Differentiated adipocytes were treated with BA (10 μM) for 16 h prior to insulin (50 nM) stimulation, then cellular glucose uptake was determined. The PPARγ agonist rosiglitazone (BRL, 0.1 μM for 16 h) served as positive control for increased glucose uptake. Values are means ± SD expressed as fold relative to the respective untreated control (cont). **P < 0.01, ***P < 0.001 (one-way ANOVA followed by Bonferroni *post hoc* test).

PPARγ agonists increase glucose uptake in adipocytes overexpressing a PPARγ dominant negative form^55^ and SR1664, a PPARγ ligand without activation property, improves insulin-stimulated glucose disposal in adipose tissue^13^, suggesting that PPARγ ligands ability to increase glucose uptake is dissociated from their transactivation property. As already described^56^, we observed that BA increased basal glucose uptake in 3T3-L1 adipocytes (Fig. 8), an effect additional to that of insulin. This result emphasizes an insulin-independent action of BA on glucose uptake.

In addition to PPARs, BA is known to interact with several biological targets and different metabolic pathways, such as inflammation and NF-kB (or p38/ERK MAPK) pathway, thereby inhibiting the endotoxin stimulated phosphorylation cascade (LPS-induced IkBα phosphorylation), and in turn IL-6 expression and prostaglandin E_2_ and COX-2 production in human peripheral blood mononuclear cells^35, 36^. Moreover, BA is an agonist of G-protein coupled receptor (TGR5)^39^, thus increasing the mitochondrial activity, and inhibits enzymes related to carbohydrates and lipids metabolism, such as pancreatic lipase and α-amilase^33^, protein tyrosine phosphatase 1B (PTP1B)^37^, glycogen phosphorylase and α-glucosidase^57^, and DGAT1^33, 58^. BA administration to mice fed high fat diets decreases plasma glucose levels, increases insulin levels, protects against hepatocellular steatosis, ameliorates hyperglycaemia and dyslipidaemia, prevents abdominal fat accumulation and decreases body weight^33^. Finally, it is well known the cytotoxic activity of BA to over 20 cancer cell lines probably triggers apoptosis^34^. Importantly, BA is much more cytotoxic to cancer cells than to normal cells, such as skin fibroblasts, peripheral blood lymphocytes and melanocytes^34^. This evidence together confirms that the natural compound BA may be a promising therapeutic agent in the treatment of different pathologies.

### Concluding Remarks

In the search for alternative PPAR modulators for the treatment of metabolic syndrome and type 2 diabetes, natural products have been shown to provide a promising pool of structures for drug discovery, especially those originating from traditionally used medicinal plants. In this work, an affinity-based bioassay was found to be effective for selecting PPAR ligands from a dried extract of an African plant. Among the ligands, we identified betulinic acid, which is already known for its anti-inflammatory, anti-tumour and antidiabetic properties, as a PPARγ and PPARα antagonist. ITC experiments confirmed its affinity towards PPARγ, and the crystal structure of the complex of BA with PPARγ shed light, at the molecular level, on its antagonism towards PPARγ. BA is the first PPARγ and PPARα antagonist whose crystal structure has been solved. The crystal structure revealed that BA does not interact with Y473 through H-bonds, but it instead forms a H-bond network with the receptor involving Y327 and S289, similarly to the known PPARγ antagonist SR1664. These results indicate a unique mode of binding of this type of antagonists, even though the BA central scaffold is different from the amino-carbonyl indole of SR1664. This structure may be helpful for designing a new class of synthetic antagonists with maintained anti-diabetic properties and fewer side effects. Moreover, another BA molecule can be modelled at a second alternate site of PPARγ LBD, which is usually occupied by partial agonists. It may be speculated that this second molecule may play a role of activity modulation at high ligand concentrations. The 3T3-L1 cell differentiation assay showed that BA inhibits the adipogenesis and down-regulates or does not affect the expression of a series of adipogenic markers. Moreover, the MC3T3-E1 cell differentiation assay showed that BA promotes osteogenesis and up-regulates the expression of osteogenic markers. Finally, the 2-deoxy-D-glucose uptake assay showed that BA improves the basal glucose uptake in 3T3-L1 cells up to 50%. In light of these results, BA is an interesting candidate for the treatment of type 2 diabetes and bone diseases, with the potential to decrease side effects.

## Methods

### Materials

HIS-Select^®^ Spin Columns Ni^2+^-nitriloacetic acid columns were from Sigma Aldrich. *D*. *bipindensis* stem bark were collected in Cameroon in July 2009 and July 2011 in the camps of Abing. The plant was identified at the Cameroon National Herbarium, Yaoundé by the Cameroonian botanist Mr Nana; a voucher specimen (no. BWPV 08) was also deposited at the Department of Drug Sciences of the University of Pavia. Stem bark was dried in the dark, in a ventilated room at 25–30 °C, then grounded and the powder stored at −20 °C. A DME extract of *D*. *bipindensis* stem bark was prepared, and the secondary acidic metabolites 4-hydroxy-5-methyl-coumarin, canaliculatin, plumbagin, ismailin and betulinic acid were isolated^22^. The chemical structures are reported in Figure S1. Plumbagin and betulinic acid, commercially available, were purchased from Sigma and used as standard while canaliculatin, ismailin and 4-hydroxy-5-methyl-coumarin were used as isolated (^1^H-NMR purity).

### Solution preparation

The three known ligands were dissolved in DMSO (1 mM) and further dilutions were carried out in water (2.5 × 10^−2^ M). 20 mg of DME were solubilized in 1 ml of methanol. The suspension was vortex mixed and centrifuged. The procedure was repeated twice. The two methanol fractions were purified on a SPE-C18 cartridge to remove lipophilic material (resins, chlorofil etc.). The methanol was collected and evaporated to dryness. 15 mg of the dried powder were then solubilized in 1.5 ml methanol and 1 ml of this solution was diluted to 10 ml with water (approximately 100 μg/ml).

1 mg of standard plumbagin was solubilized in 1 ml methanol. This solution was diluted with water (final concentration 500 ng/ml). 1 mg of standard betulinic acid was solubilized in 1 ml methanol and the solution was diluted 1:100 with water (final concentration 10 μg/ml). 1 mg aliquots of purified 4-hydroxy-5-methyl-coumarin, canaliculatin or ismailin were dissolved in 1 ml methanol and the solutions were further diluted 1:10 with water (100 μg/ml).

### Bioaffinity cartridges preparation

The His-tag receptor was immobilized on HIS-Select Spin Column through non covalent binding between His-tag protein residues and the nickel complex of the sorbent. SPE tubes were processed individually using a syringe and an adapter to provide positive pressure to force the liquid through the tube. The column was first conditioned with 1.8 ml 10 mM phosphate buffer pH 7.5. An aliquot of 200 μL of PPARγ solution in 20 mM TRIS buffer pH 8, 150 mM NaCl, 40% glycerol, 1 mM TCEP (approximately 665 μg protein) was diluted to 400 μL with phosphate buffer (10 mM, pH 7.5) and flushed through the cartridges three times. The column was then washed with 1 ml of the same buffer. The amount of immobilized receptor (250 μg of receptor, immobilization yield ≈ 38%) was estimated by Bradford assay^59^. Non-specific interaction of ligands to the support was measured using control supports which were prepared by following the same method except that no receptor was added during the immobilization step.

### Bioaffinity assay

The bioaffinity assay was performed in parallel on the receptor cartridge (RC) and on the control cartridge (CC). The bioaffinity assay was carried out during assay validation with known ligand and to rank secondary metabolites contained in the DME. The solutions containing each ligand alone or in mixture were prepared as previously described. A common procedure was followed.

300 μl of ligand solution was gently flushed through the receptor cartridges. Sample loading was followed by the elution with 600 μl phosphate buffer. The eluate was collected in seven fractions (F1–F7) of 110 μl each. After each experiments the cartridges were regenerated with 1.8 ml phosphate buffer. The bioaffinity assay was performed in parallel on the receptor cartridge and on the control cartridge. Each fraction was analysed by HPLC using *method 1* for known ligand and *method 2* for secondary metabolites affinity ranking. From each chromatogram the areas of each ligand in the seven fraction eluted from the receptor column (A_i_) and the areas of the same ligands in the seven fraction eluted from the control column were determined. In order to assess the elution profiles of the analytes during the bioaffinity assay, a graphical representation was derived by reporting on the y-axis the percentage of eluted analyte (Ai/At)*100 and on x-axis the cumulative elution volume. The breakthrough curves were analysed with a polynomial model to fit the chromatographic data.

### Chromatography

Chromatographic analyses were carried out on a HP 1100 system equipped with a photodiode array detector (Agilent Technologies, Santa Clara, CA, USA); X-Bridge (Waters, MA, USA) C18 column (5 µm, 150 × 4.9 mm i.d.). Two methods were developed for the analysis of the acidic metabolites of DME extract and of the three known ligands (Method 1 and Method 2, respectively).

*Method 1*. Mobile phase MeOH-H_2_O-0.02% TFA in gradient mode: 5% to 100% of MeOH in 50 min, 100% of MeOH for 10 min; flow rate: 1 ml/min; injection volume 20 µl; detection at 217 and 254 nm. Retention times were: 4OH-5CH_3_-coumarine 32.56 min; canaliculatin 34.61 min; plumbagin 35.58 min; ismailin 42.61 min; betulinic acid 50.99 min.

*Method 2*. Mobile phase MeOH-H_2_O-0.05% phosphoric acid in gradient mode. Analyses were realized with a gradient elution from 75% of methanol + 0.05% H_3_PO_4_ to 95% of methanol + 0.05% H_3_PO_4_ in 15 minutes. Retention times for the three reference compounds were: low affinity (compound A) 6.71 min; medium affinity (compound B) 8.46 min; high affinity (compound C) 9.64 min.

### PPAR Protein Expression and Purification

PPARγ LBD was expressed as N-terminal His-tagged proteins using a pET28 vector and then purified as previously described^60^. Briefly, freshly transformed *E*.*coli* BL21 DE3 were grown in LB medium with 30 μg of kanamycin/ml at 310 K to an OD of 0.6. The culture was then induced with 0.2 mM isopropyl-β-D-thio-galactopyranoside and further incubated at 291 K for 20 h. Cells were harvested and resuspended in a 20 ml/liter culture of Buffer A (20 mM Tris, 150 mM NaCl, 10% glycerol, 1 mM Tris 2-carboxyethylphosphine HCl (TCEP), pH 8) in the presence of protease inhibitors (Complete Mini EDTA-free; Roche Applied Science). Cells were sonicated, and the soluble fraction was isolated by centrifugation (35,000 × g for 45 min). The supernatant was loaded onto a Ni^2+^-nitrilotriacetic acid column (GE Healthcare) and eluted with a gradient of imidazole 0–500 mM in Buffer A (batch method). The pure protein was identified by SDS PAGE. The protein was then dialyzed over buffer A to remove imidazole, and it was cleaved with thrombin protease (GE Healthcare) (10 units/mg) at room temperature for 2 h. The digested mixture was reloaded onto a Ni^2+^-nitriloacetic acid column to remove His tag and the undigested protein. The flow-through was dialized with buffer B (20 mM Tris, 10% glycerol, 1 mM TCEP, pH 8) to remove NaCl and then loaded onto a Q-Sepharose HP column (GE Healthcare) and eluted with a gradient of NaCl 0–500 mM in Buffer B with a BioLogic DuoFlow FPLC system (Bio-Rad Laboratories, Italy). Finally, the proteins were purified by gel-filtration chromatography on a HiLoad Superdex 75 column (GE Healthcare) and eluted with Buffer C (20 mM Tris, 1 mM TCEP, 0.5 mM EDTA, pH 8). The proteins were then concentrated at 8 mg/ml using Amicon centrifugal concentrators with a 10 kDa cutoff membrane (Millipore, USA).

### Crystallization and Data Collection

Crystals of apo-PPARγ were obtained by vapor diffusion at 18 °C using a sitting drop made by mixing 2 μL of protein solution with 2 μL of reservoir solution (0.8 M Na Citrate, 0.15 M Tris, pH 8.0). The crystals were soaked for one week in a storage solution (1.2 M Na Citrate, 0.15 M Tris, pH 8.0) containing the ligand BA (0.5 mM). The ligand dissolved in DMSO (50 mM) was diluted in the storage solution so that the final concentration of DMSO was 1%. The storage solution with glycerol 20% (v/v) was used as cryoprotectant. Crystals (0.2 × 0.2 mm) of PPARγ/BA belong to the space group *C2* with cell parameters shown in Table 1.

### Structure Determination and Refinement

X-ray data set were collected at 100 K under a nitrogen stream using sinchrotron radiation (beamline BM14U at ESRF, Grenoble, France). The collected data were processed using the programs XDS and XSCALE^61^. Structure solution was performed with AMoRe^62^, using the coordinates of PPARγ/LT175R^51^ (PDB code 3D6D) as the starting model. The coordinates were then refined with CNS^63^. All data between 50.00 and 2.0 Å were included in the refinement. A final step of refinement was performed with the software Phenix^64^. The statistics of crystallographic data and refinement are summarized in Table 1.

### Microcalorimetry

ITC reverse titration experiment was performed at 25 **°**C using a MicroCal ITC200 microcalorimeter (MicroCal Inc., Northampton, MA). PPARγ was extensively dialyzed against the buffer Hepes (20 mM, pH 8.0), TCEP (1 mM), with Amicon Ultra filters, and the final exchange buffer was then used to dilute the ligand stock solution (50 mM in DMSO). DMSO was added to the protein solution at the same percentage of the ligand solution (0.1%). Samples were centrifuged before the experiments to eliminate possible aggregates. Protein and ligand solutions were degassed before use. The reverse titration was performed with the protein solution (500 μM) injected into the cell containing the ligand (50 μM). The titrations involved 19 injections of 2 μL at 180 s intervals. The syringe stirring speed was set at 1000 rpm. Reference titrations of the protein into buffer were used to correct for heats of dilution. The thermodynamic data were processed with Origin 7.0 software provided by MicroCal. The ΔH values were measured for each titration, and fitting the binding isotherms with a one-site binding model yielded the values of the association constant (K_a_). From the Gibbs**−**Helmholtz equation the change of entropy (ΔS) was also calculated. The inflection point in the calorimetric isotherm gave the stoichiometry value n, indicating the ligand/protein ratio of the binding. To correct for any discrepancies in the baseline outlined by the software, a manual adjustment was performed.

### Theoretical computation of BA

The molecular structure of BA was optimized at the PM6 level of theory by using the software package MOPAC^65^. The electronic densities of the orbitals were computed at the PM6 level of theory.

### Biological methods

Reference compounds, media, and other cell culture reagents were purchased from Sigma–Aldrich (Milan, Italy).

### Plasmids

The expression vectors expressing the chimeric receptors containing the yeast Gal4 DNA binding domain fused to the human PPARα or PPARγ LBD, and the reporter plasmid for these Gal4 chimeric receptors (pGal5TKpGL3) containing five repeats of the Gal4 response elements upstream of a minimal thymidine kinase promoter that is adjacent to the luciferase gene were described previously^66^.

### Cell culture and transfections

Human hepatocellular liver carcinoma cell line HepG2 (Interlab Cell Line Collection, Genoa, Italy) was cultured in Minimum Essential Medium (MEM) containing 10% heat-inactivated fetal bovine serum, penicillin G (100 U mL^−1^), and streptomycin sulfate (100 μg mL^−1^) at 37 °C in a humidified atmosphere of 5% CO_2_. For transactivation assays, 10^5^ cells per well were seeded in a 96-well plate and transfections were performed after 24 h with K2 Transfection System (Biontex Laboratories GmbH), according to the manufacturer’s guidelines using 0.20 μg/well of DNA. Cells were transfected with expression plasmids encoding the fusion protein Gal4–PPARα LBD or Gal4–PPARγ LBD, pGal5TKpGL3, and pCMVβgal to normalize the transfection efficacy. 24 h after transfection, medium was replaced with fresh complete growth medium supplemented with the indicated ligands and reference compounds in triplicate. After further 24 h of incubation, luciferase activity in cell extracts was determined by a luminometer (VICTOR^3^ V Multilabel Plate Reader, PerkinElmer) and normalized for β-galactosidase activity as described previously^67^. Fold induction activity was calculated and plotted using GraphPad Prism5 software.

### Cell culture for staining tests and RT-PCR analysis

3T3-L1 cells (from ATCC) were routinely cultured in Dulbecco’s modified Eagle’s medium with 4 mM l-glutamine, 4.5 g/liter glucose, 0.11 g/liter sodium pyruvate, and supplemented with 10% fetal bovine serum plus antibiotics. Two days after confluence, preadipocytes were staged to differentiate by changing the medium to one containing the induction mixture (0.1 μM dexamethasone, 500 μM 3-isobutyl-1-methylxanthine, and 174.5 nM insulin). After 48 h (d0), the medium was removed and replaced by Dulbecco’s modified Eagle’s medium containing 174.5 nM insulin. BA was added after the induction phase of 3T3-L1 adipocyte differentiation (d0) in order to avoid any interference on the initiation of the genetic program that drives adipocytes differentiation and the media was renewed every other day.

MC3T3-E1 cells (from ATCC) were cultured in α-MEM to confluency and then cultured in differentiation medium (α-MEM supplemented with 200 μM ascorbic acid and 10 mM b-glycerophosphate) for 14 days, which was replaced every 3 days. The cells were treated with BA throughout the differentiation.

### Oil Red O staining

3T3-L1 cells were washed with PBS and fixed with 4% formaldehyde solution for 20 minutes, then washed again and stained with 0.35% Oil Red O solution in 60% isopropanol for 20 minutes. Then, cells were washed with sterile double distilled water, and photographs were taken. The stain from the cells was eluted using 100% isopropanol and the absorbance of the eluted stain was read at 490 nm.

### Alizarin Red S staining

MC3T3-E1 cells were washed with PBS and fixed with 4% formaldehyde solution for 20 minutes, then washed again and stained with Alizarin Red S (2% solution) for 20 minutes. Then, cells were washed with sterile double distilled water, and photographs were taken. Quantification of staining was performed as previously described^68^.

### 2-deoxy-D-glucose uptake assay

Glucose uptake activity of 3T3-L1 adipocytes was measured by the chemiluminescent assay^69^ using Glucofax kit as described by the manufacturer (Yelen, Ensues la Redonne, France).

### Real Time PCR Analysis

Total RNA was extracted using a Nucleospin RNA kit (Macherey-Nagel, Hoerdt, France), cDNA was synthesized from 0.5 μg of RNA using Moloney murine leukemia virus reverse transcriptase (Invitrogen). Real time PCRs were performed on the LightCycler 480 instrument (Roche Applied Science) using the Eva Green MasterMix (Euromedex, Souffelweyersheim, France). The comparative Ct method (2^−(ΔΔCT)^) was used to calculate the relative differences in mRNA expression. The acidic ribosomal phosphoprotein P0 (*Rplp0*) was used as housekeeping gene. Primers sequences are available upon request.

### Statistical analysis

All transfection experiments were performed in triplicate and were repeated at least twice with similar results. The results were expressed as mean ± SEM. The responses to agonists or antagonists were calculated as a percentage compared to control, which was set to 100%.

## Electronic supplementary material

Supplementary data
