# Supplementary material for: Betulinic acid is a PPARγ antagonist that improves glucose uptake, promotes osteogenesis and inhibits adipogenesis
Source: Sci Rep. 2017 Jul 18;7:5777. doi: 10.1038/s41598-017-05666-6 (PMC5516003; doi:10.1038/s41598-017-05666-6)
Supplement: Supplementary file 1 — Supplementary data [file 41598_2017_5666_MOESM1_ESM.pdf]

Betulinic acid is a PPARγ antagonist that improves glucose uptake, promotes osteogenesis and inhibits adipogenesis

Gloria Brusotti[1], Roberta Montanari[2], Davide Capelli[2], Giulia Cattaneo[1], Antonio Laghezza[3], Paolo Tortorella[3], Fulvio Loiodice[3], Franck Peiretti[4], Bernadette Bonardo[4], Alessandro Paiardini[5], Enrica Calleri[1,*] and Giorgio Pochetti[2,*]

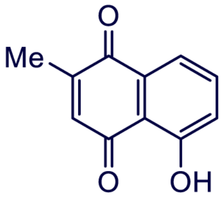

Plumbagin

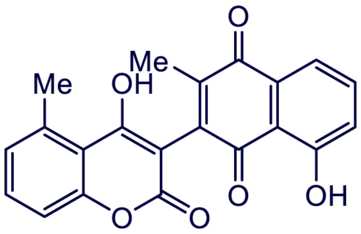

Canaliculatin

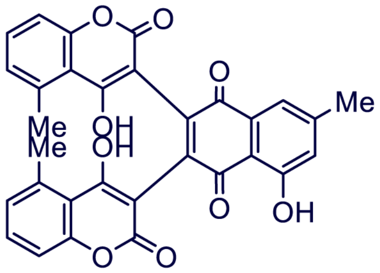

Ismailin

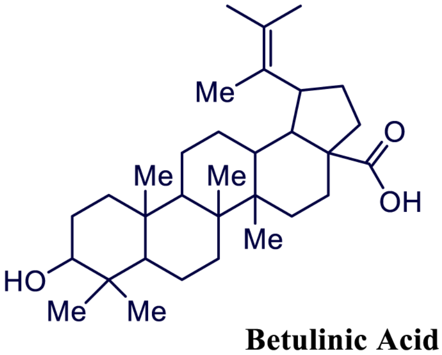

Betulinic Acid

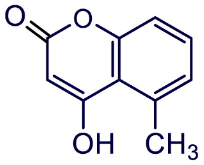

4-hydroxy-5-methyl-coumarin

Supplementary Figure S1: Structure of secondary metabolites contained in *Diospyros bipindensis*.

	Compound	Acronym	Config.	PPARγ	
				EC_{50} (nM)	E_{max} (%)
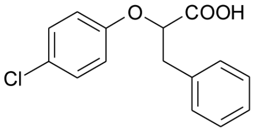	$C_{15}H_{13}ClO_3$ 276.72	(A)	*S*	2700±18 0	58±6
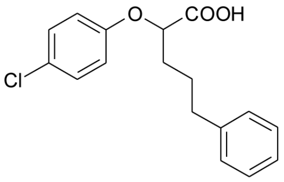	$C_{17}H_{17}ClO_3$ 304.78	(B)	*S*	320±10	66±4
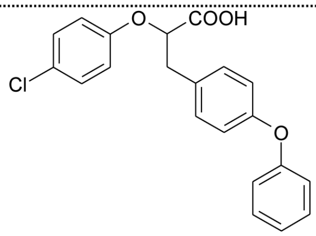	$C_{21}H_{17}ClO_4$ 368.82	(C)	*S*	26±4	68±6

Supplementary Figure S2: Three known ligands used as reference in the bioaffinity experiments.

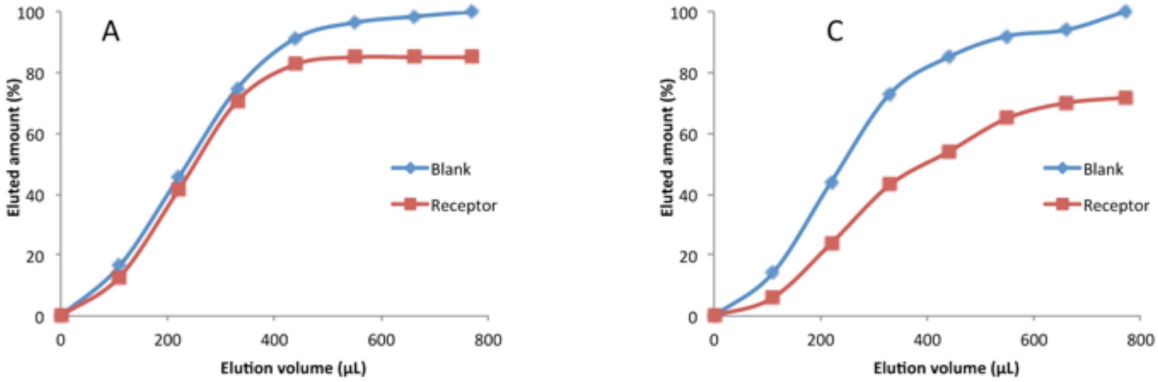

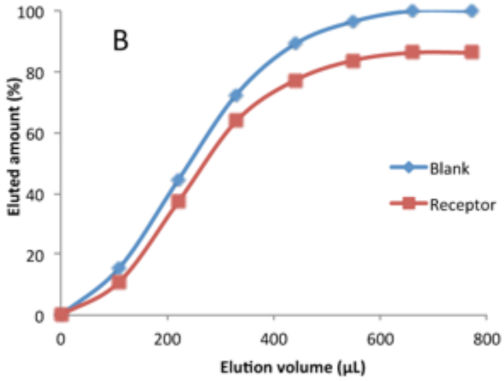

D

Δ elution volume	Mix (µL)	Alone (µL)
A	5	25
B	13	68
C	60	92

Supplementary Figure S3: The experimental breakthrough elution profiles for the three reference analytes.

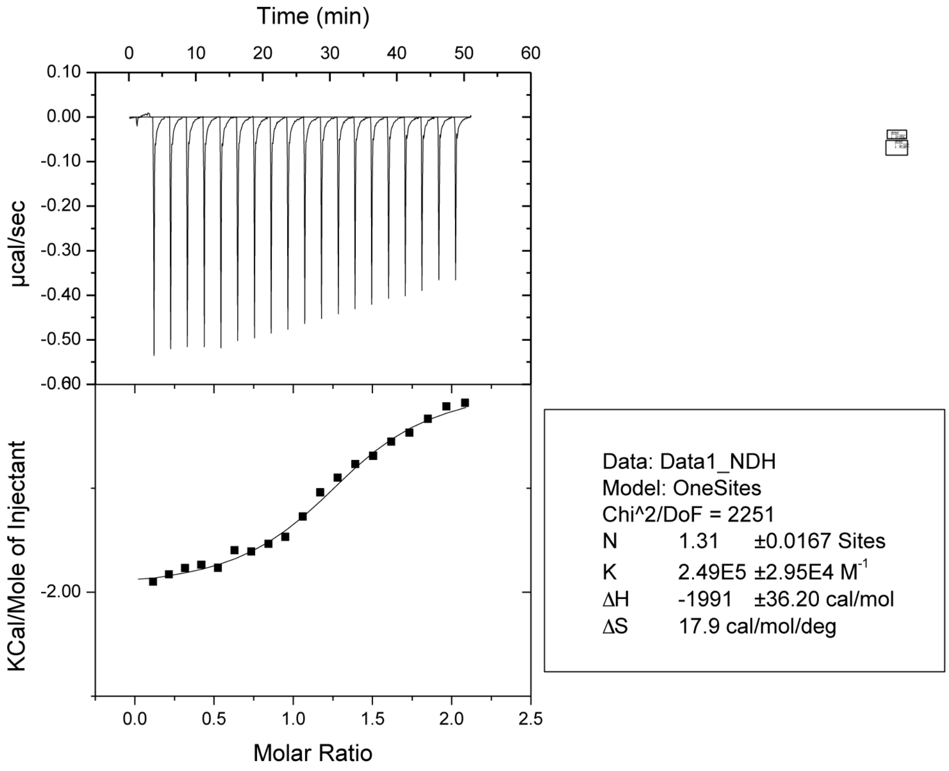

Supplementary Figure S4: Inverse titration of BA (50 μM) with PPARγ (500 μM). The upper panel shows the raw data; the lower panel shows the corresponding binding isotherm, fitted according to the "one binding site" model. The thermodynamic parameters (K_d, ΔH and ΔS) are indicated in the box.

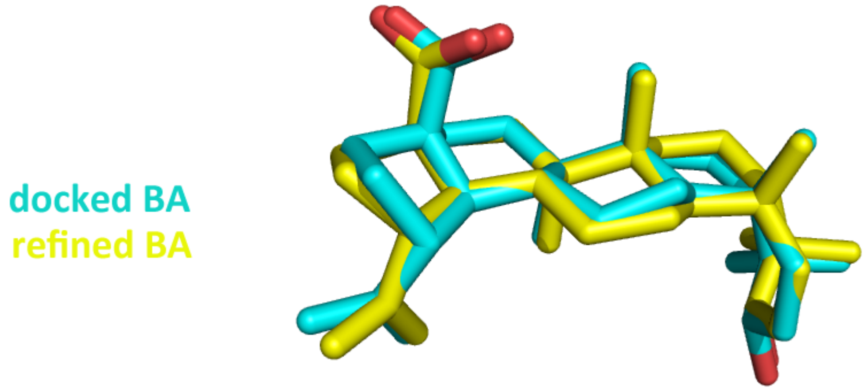

Supplementary Figure S5: Superposition of the docked ligand with the refined ligand in the primary site of PPARγ LBD.

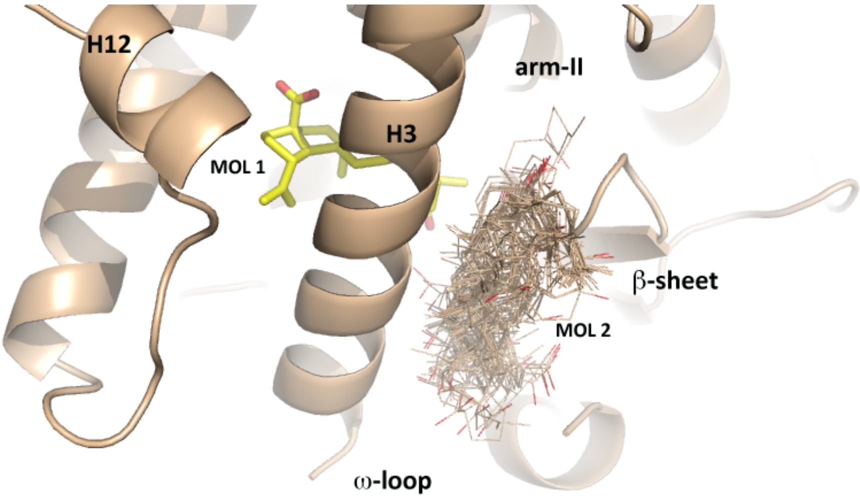

Supplementary Figure S6: Set of the energetically favourable poses of a second molecule of BA in the secondary site of PPARγ LBD.

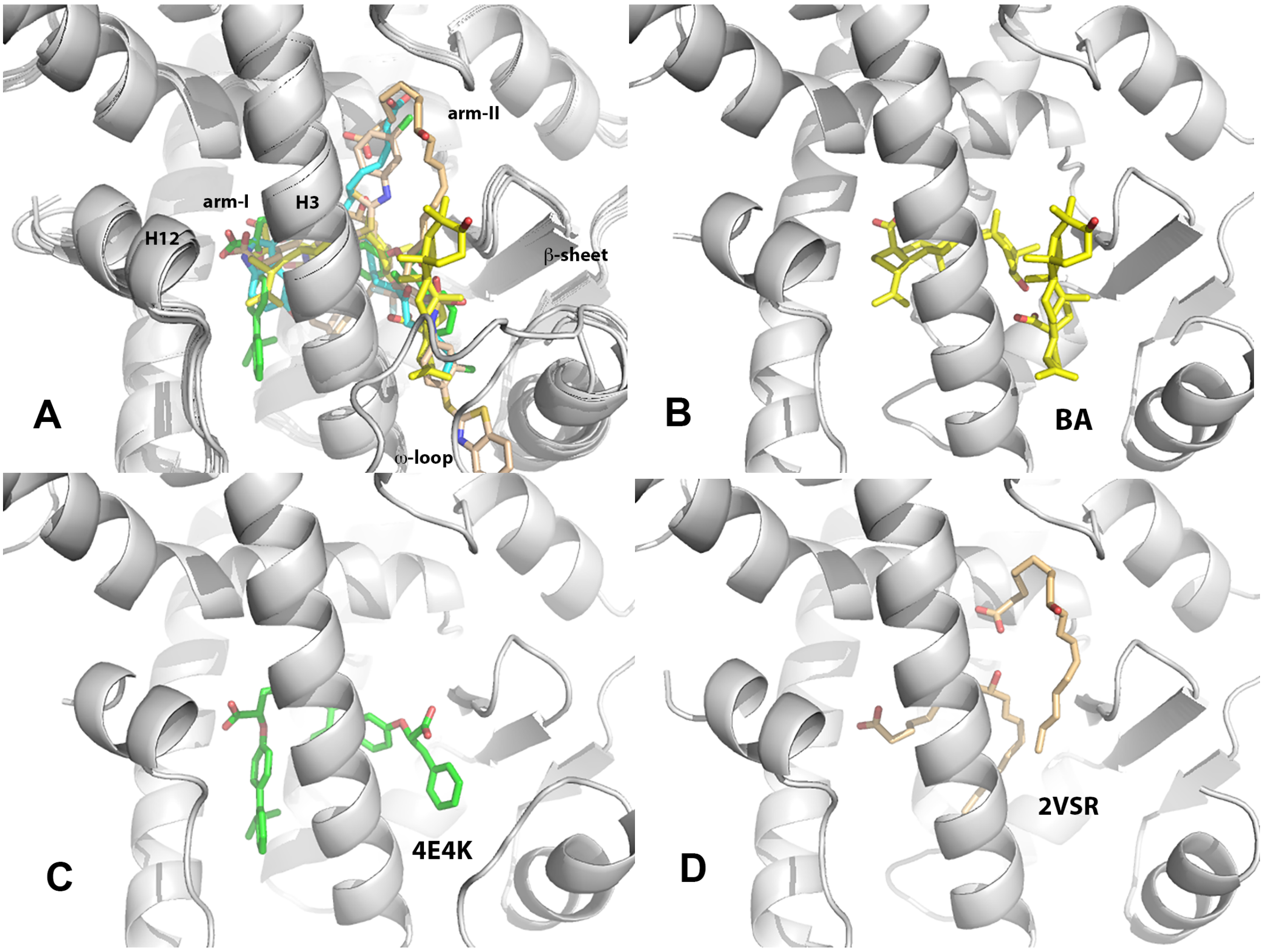

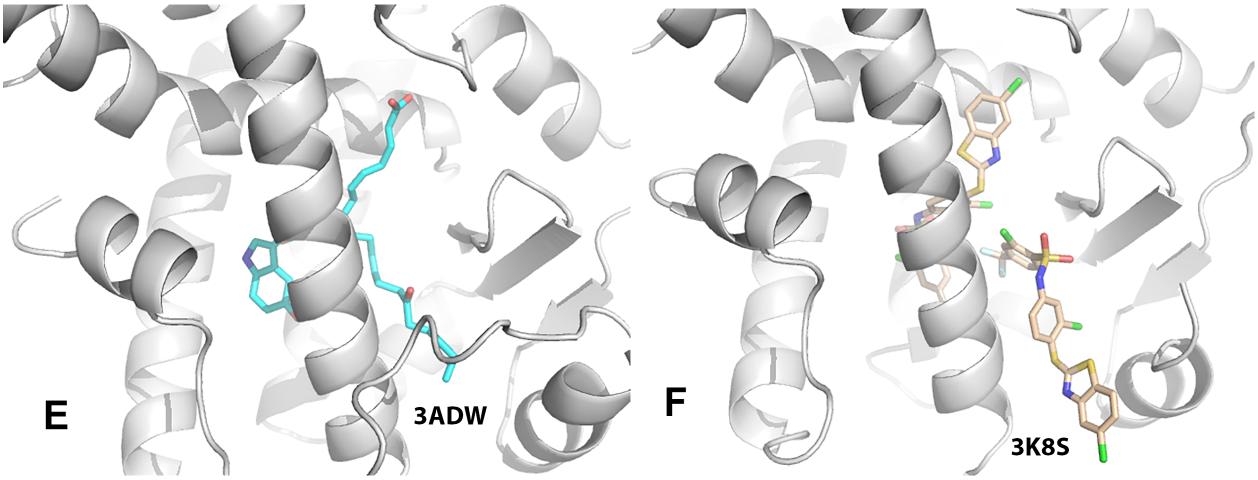

Supplementary Figure S7: Comparison among five pdb structures of PPARγ complexes with two simultaneously bound molecules: (A) superposition of the five ligands in the PPARγ LBD; PPARγ complexes with (B) betulinic acid, (C) 2-(Aryloxy)-3-phenylpropanoic acid JO21 (pdb 4E4K) , (D) oxidised fatty acid 9-(S)-HODE (pdb 2VSR), (E) 5-methoxy-indole acetate and 15-oxo-eicosatetraenoic acid (pdb 3ADW) and (F) T2384 (pdb 3K8S).

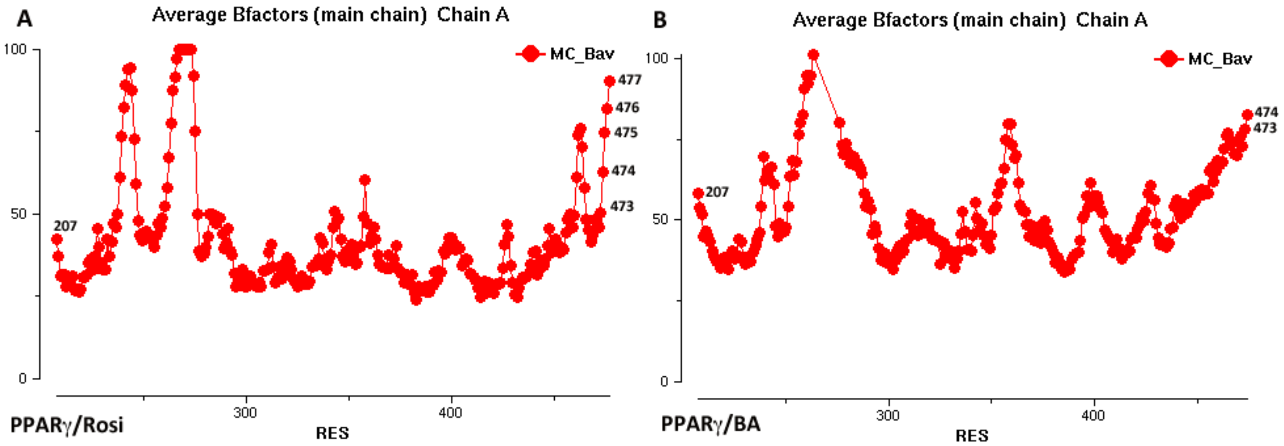

Supplementary Figure S8: Comparison of the helix12 B factors of the structure with the full agonist rosiglitazone (pdb code 2PRG) with those of PPARγ/BA.

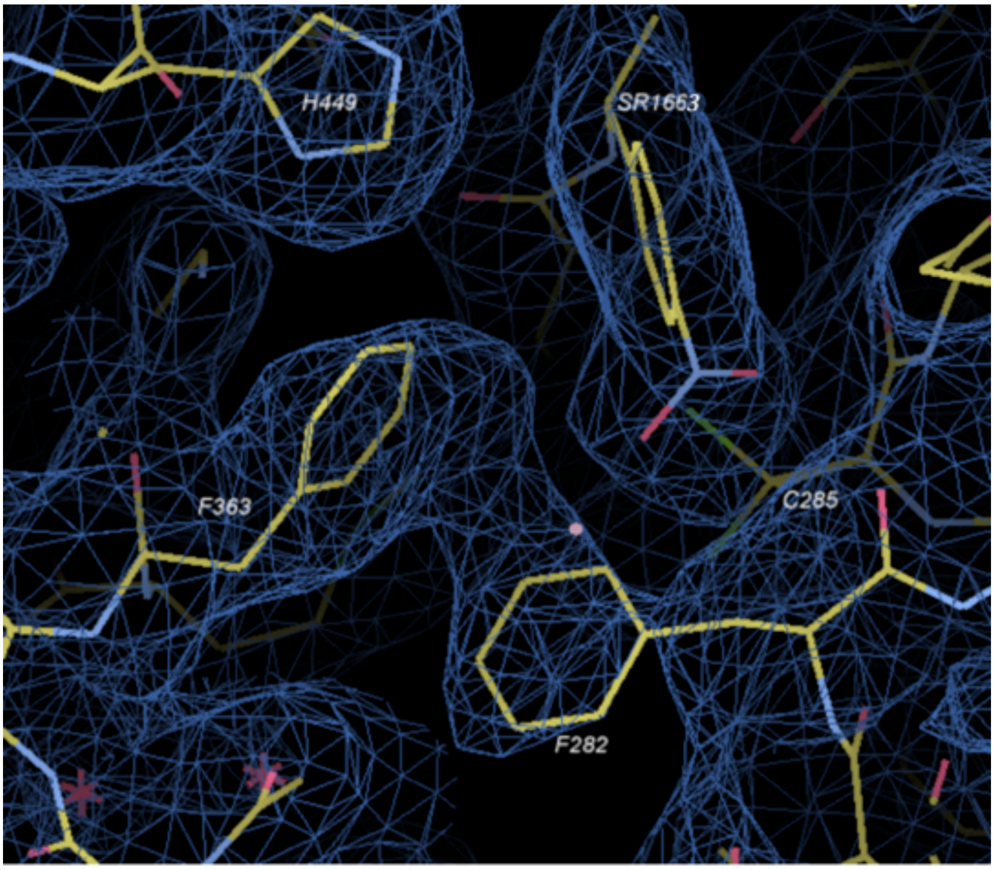

Supplementary Figure S9: 2Fo-Fc electron density maps reveals a possible different conformation of the F282 side-chain in the complex PPARγ/SR1663 (pdb 4R6S).
